# Supplementary material for: Functionally defined white matter of the macaque monkey brain reveals a dorso-ventral attention network
Source: eLife. 2019 Jan 2;8:e40520. doi: 10.7554/eLife.40520 (PMC6345568; doi:10.7554/eLife.40520)
Supplement: Supplementary file 1. — For each functional ROI used during tracking procedure we report the behavioral task used for the functional mapping, the AP position of stereotactic coordinates in AC coordinate (center of ROI), and the anatomical AP position of the corresponding areas in Saleem and Logothetis (2007). [file elife-40520-supp1.docx]

|  | **Task** | **Logothetis**  **Saleem** | **M1**  **functional** | **M2**  **functional** |
| --- | --- | --- | --- | --- |
| **STS** |  |  |  |  |
| PITd | target/distractor random dot patterns | +2.5 (TEO) | +3 | -2.5 |
| **IPS** |  |  |  |  |
| LIP | target/distractor random dot patterns | +1 | -0.75 | -6.25 |
| **AS** |  |  |  |  |
| FEF | single dot at 9 spatial locations | +27 | +30 | +21 |
| **visual** |  |  |  |  |
| V4d | vertical and horizontal checkerboard wedge | -1 | -2.5 | -4.25 |
| V4v | vertical and horizontal checkerboard wedge | -3.5 | -4 | -6.5 |

**Supplementary file 1. Functional ROI mapping**. For each functional ROI used during tracking procedure we report the behavioral task used for the functional mapping, the AP position of stereotactic coordinates in AC coordinate (center of ROI), and the anatomical AP position of the corresponding areas in (Saleem & Logothetis, 2007).
